# Greater brain volumes are activated when performing simple calculating tasks in persons accustomed to lower altitudes than those to higher altitudes

**DOI:** 10.1002/brb3.148

**Published:** 2013-06-11

**Authors:** Yan L Jiang, Willmann Liang, Hong Yao, David T Yew

**Affiliations:** 1 Department of Radiology, Kunming Medical UniversityKunming, Yunnan, China; 2 Brain Research Centre, Institute of Chinese Medicine, The Chinese University of Hong KongHong Kong, China; 3 Centre for Emerging Infectious Diseases, The Chinese University of Hong KongHong Kong, China

**Keywords:** Altitude, brain, fMRI, simple calculation

## Abstract

Chronic exposure to a hypoxic environment results in a number of physiological changes such as cardiac arrhythmia and pulmonary edema. We hereby studied the variations in activation of brain areas during simple calculation tasks between individuals originating from different altitudes. Two groups of subjects, one from 1700 m above sea level (lowlanders) and the other one from at least 3000 m above sea level (highlanders), performed a simple calculation task by heart. The fMRI were taken and horizontal, sagittal, and coronal sections were analyzed to identify activated brain areas. Both lowlanders and highlanders performed the calculation task successfully. Horizontal sections revealed similar activated areas in the deep and anterior part of the right parietal lobe of both lowlanders and highlanders. In the highlanders, coronal and sagittal sections showed lower activities. Smaller brain volumes were activated in the highlanders as shown by the computer brain templates, with fewer total voxels recorded than in the lowlanders (*P* = 0.003). Computerized comparison of overall active brain regions between lowlanders and highlanders also revealed that smaller brain regions were activated. The results showed that while all subjects completed the task successfully, the highlanders did so using smaller brain regions than the lowlanders.

## Introduction

It is well known that hypoxia induces mountain sickness, pulmonary edema, cardiac arrhythmia, cerebral hypoxia, and immunosuppression (Bailey and Davies 1997). Although there were claims that exercise in the high altitude improved performance at sea level, the evidence was not unequivocal (Bailey and Davies 1997). Exposure acutely to moderate altitude (2000–3000 m) would increase ventilation, increase heart rate, decrease stroke volume, reduce plasma volume, and lower maximal aerobic power by 15–20%. After several weeks, an increase in volume of red blood cells and aerobic power was observed (Saunders et al. 2009). However, whether the increase in red blood cell volume would increase performance at sea level was not evident (Saunders et al. 2009).

Upon hypoxia, the EEG of the brain displayed spectrum and phase instability (Balioz and Krivoshchekov 2012), which was apparent with only 20 min of acute hypoxia (Schellart and Reits 2001). A significant increase in power of theta and alpha bands was featured during hypoxic stress (Papadelis et al. 2007). Increased physical training assisted in increased resistance to acute hypoxia, for example, facilitated lipid peroxidation and antioxidation enzymes (Sazontova et al. 2012), induced rises of malondialdehydes and advanced oxidation products, arterial oxygen hemoglobin desaturations, ferric-reducing antioxidant power values decreased with alpha-tocopherol/triglyceride ratio (Pialoux et al. 2006).

In spite of studies on training normoxic individuals on high altitude and expecting improved performances on returning to normoxic environment, there have been no studies on the comparison of hypoxia-adapted individuals at normoxic environment with normoxic individuals. This is a study on the simple cognitive abilities (in this case, simple mathematics by heart) comparing these two groups. For simple solving of mathematic problems, Fehr et al. (2007) attributed to the brain areas dealing with working memory and numerical knowledge, particularly with parietal areas (Dehaene et al. 1999, 2003). Functional MRI indicated bilateral activation in the horizontal part of the intraparietal sulcus and the posterior parietal lobule upon simple mental calculation without finger movement (Andres et al. 2012), which might enlist other regions of the frontal and central cortices as well (Fehr et al. 2007). If there was any anxiety in the solving of mathematical questions, the amygdala might also fire (Young et al. 2012). Noting the brain regions of concern when engaged in this particular mental task, we compared the differences in activation areas between normoxic individuals and those adapted to hypoxia, but were now residing in a normoxic environment.

## Methods

### Subjects

Two groups of students from 17 to 21 years old were enlisted. Each group had 10 students. The first group (i.e., lowlanders) had students aged between 17 and 19 who were natives of Yunnan province, China, living continuously at 1700 m above sea level. The second group (i.e., highlanders) consisted of students aged between 17 and 21 who were dwelling at 3000 m or more above sea level in the highlands. The latter group of students had come to Yunnan as students in the university just 1 month prior to this study. The project had informed consent from all the students involved and had ethical approval from the hospital and university involved.

### Simple mental task of mathematics

The students were asked to compute a short and easy mathematical question by heart after presented the question via a slide. The simple question was in the form of X × Y + Z. While the students were computing, fMRI was performed on their brains. Apart from the lowlander and the highlander groups, five controls (age matched) were employed. These latter subjects were provided with slides of different sceneries while fMRI were performed on them.

### Image processing

Processing and analysis of fMRI data was performed using the MATLAB software coupled with the Statistical Parametric Mapping 8 (SPM8) method developed by the Wellcome Department of Cognitive Neurology, University College London (http://www.fil.ion.ucl.ac.uk/spm/) as described previously (Yu et al. 2012). The steps are described briefly as follows. Images from each subject were realigned with the first scanned image to correct for head movement artefacts during the fMRI. Coregistration was then performed to give information correlating functional and structural MRI data. Structural MRIs from all subjects were coaligned to generate a statistically averaged brain template. This template was used for the individual subject to whom MRI data were registered and followed by reslicing. The resulting voxel size was 2 mm × 2 mm × 2 mm. To improve the signal-to-noise ratio, the resliced fMRI data were smoothened using a Gaussian kernel of 8 mm.

### Image analysis

The analytical method in this study was the same as that in our previous study (Yu et al. 2012). The fMRI data were estimated using the General Linear Model (GLM). For individual fMRI, a threshold *P* value of less than 0.05 (after family-wise error correction) was considered statistically significant during brain activation. For comparison between groups, a threshold *P* value of less than 0.001 (uncorrected) was considered statistically significant. Cluster sizes measuring 10 voxels were included for the analysis.

## Results

**Figure 1:**
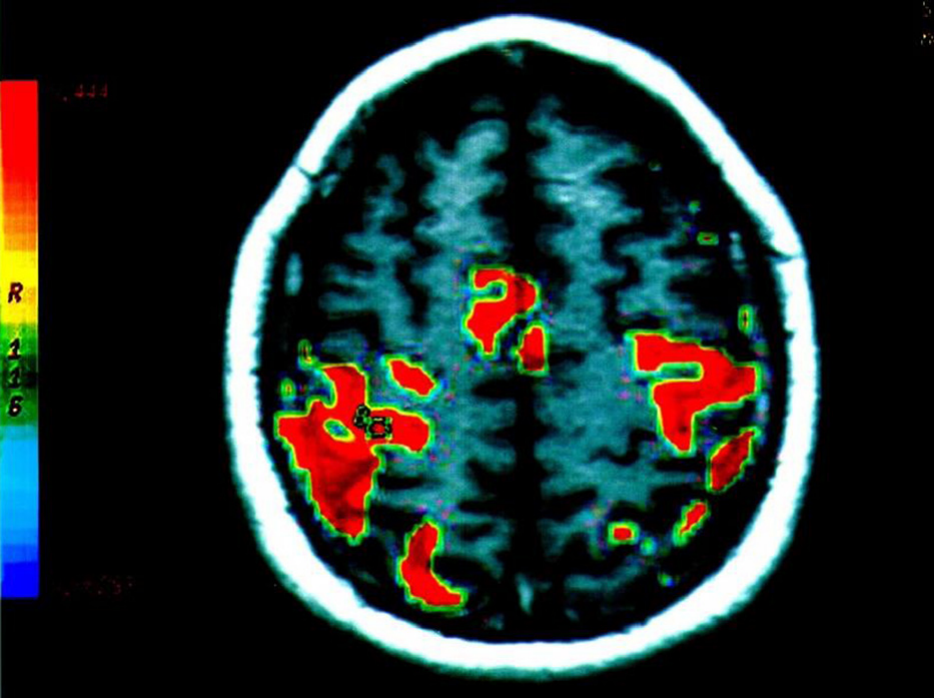
Representative fMRI of normal age-matched subjects looking at slides of scenery.

**Figure 2:**
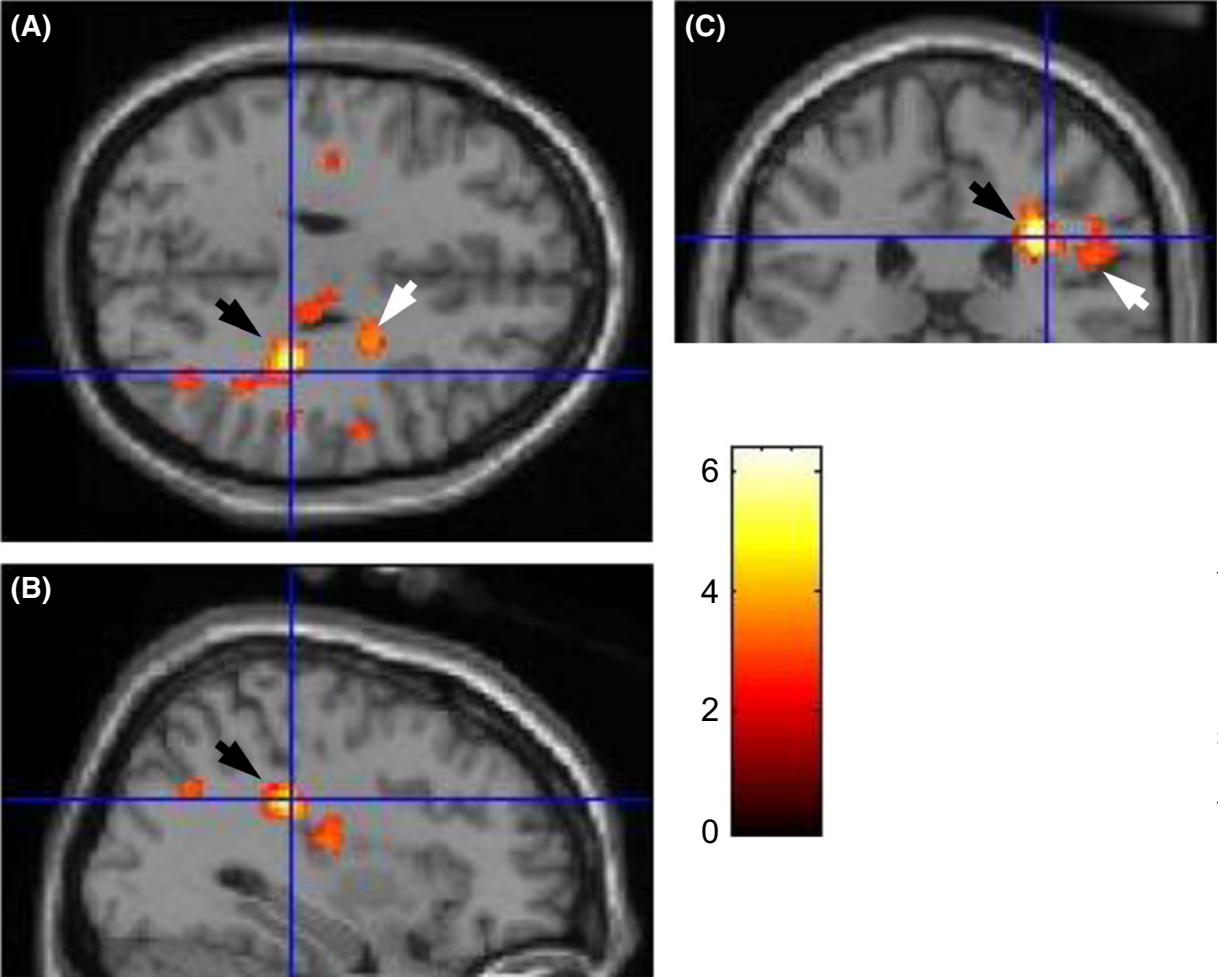
Representative fMRI of lowlanders shown in (A) horizontal, (B) lateral sagittal, and (C) coronal sections. The horizontal section revealed one high- (black arrowhead) and one low-intensity (white arrowhead) areas. The high-intensity area in the horizontal section (black arrowhead) was also revealed in the lateral sagittal section. The coronal section revealed another high-intensity area in the frontal area lateral to the lateral ventricle (black arrowhead) extending into a low-intensity area (white arrowhead).

The fMRI on control subjects looking at scenic pictures revealed positive sites on the parietal and visual areas (Fig. 1) with a small positive site around the central area (Fig. 1), whereas no positive sites were evident on the premotor area or any parts of the frontal region. On the other hand, all the experimental subjects in both the lowlander and highlander groups obtained right answers for the questions. The representative fMRI, however, revealed some differences in the lowlanders (i.e., those from 1700 m above sea level, normal inhabitants of Yunnan) and the highlanders who came into the region for study (i.e., those originally from at least 3000 m above sea level). For the normal inhabitants or lowlanders, the horizontal section showed positive high-intensity sites in the deep and anterior part of the parietal area (Fig. 2A, black arrowhead) and an insignificant low-intensity site in the posterior part of the deep frontal cortex (anterior to the motor cortex and to the right lateral ventricle) (Fig. 2A, white arrowhead). The high-intensity area (Fig. 2A, black arrowhead) was also indicated in the lateral sagittal section, which showed a high-intensity area (Fig. 2B, black arrowhead) close to the cortex. The coronal section further revealed a high-intensity area (Fig. 2C, black arrowhead) superior and in the anterior brain, in the frontal cortex anterior to the lateral ventricle. Note that this section (Fig. 2C) was cut at an angle through the frontal cortex and the anterior part of the temporal lobe. Immediately adjacent the high-intensity area in Figure 2C (black arrowhead) was a low-intensity band extending laterally toward the surface of the cortex (Fig. 2C white arrowhead). The active site in the lateral sagittal section (Fig. 2B, black arrowhead) was located posterior and close to the corpus callosum. The same parietal area was indicated in Figure 2A (black arrowhead) and Figure 2B (black arrowhead) while the activation of the frontal area was shown in Figure 2C.

**Figure 3:**
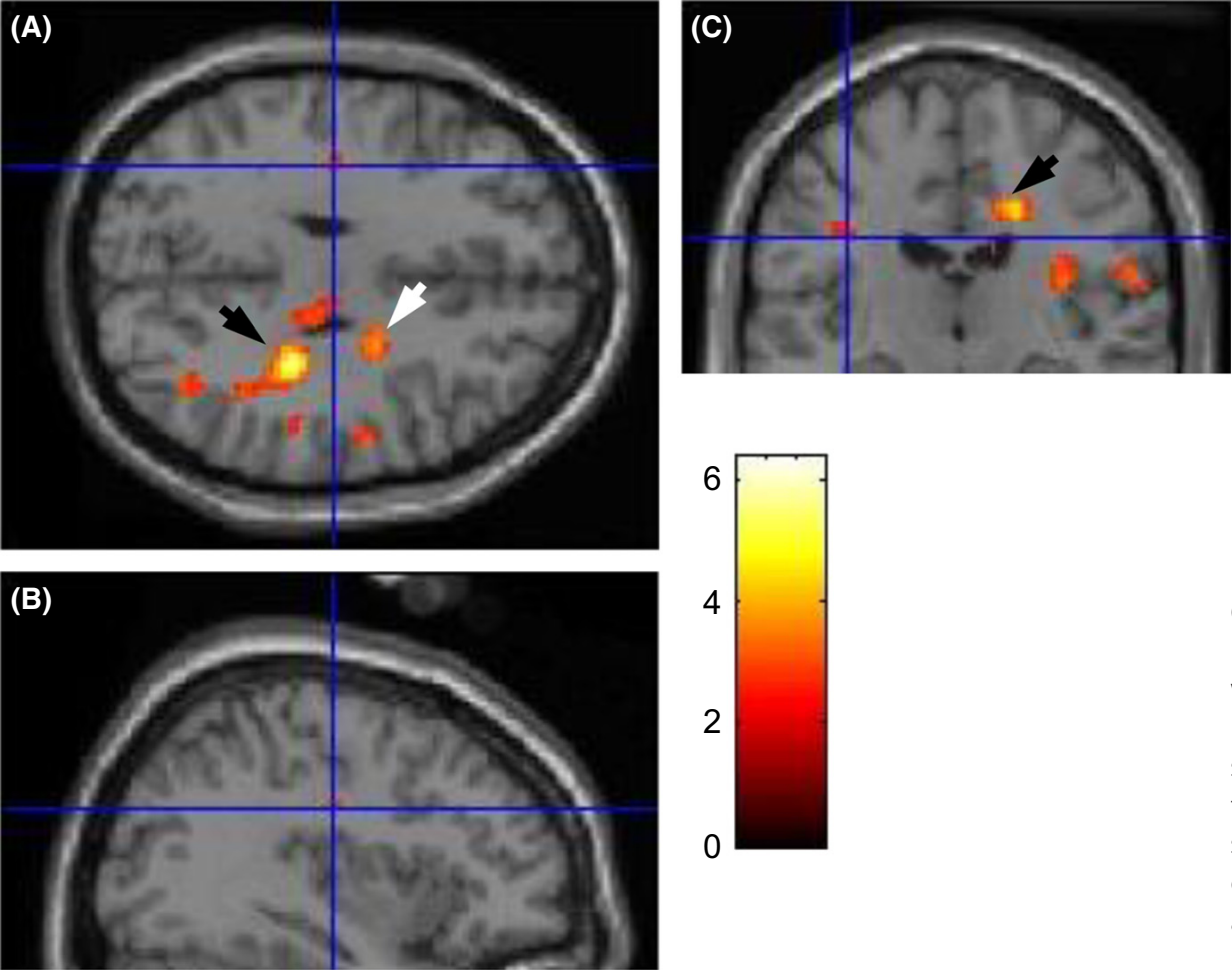
Representative fMRI of highlanders shown in (A) horizontal, (B) lateral sagittal, and (C) coronal sections. Overall fewer active areas were present when compared with the lowlanders. The horizontal section revealed active areas similar to those in the lowlanders, but the coronal and lateral sagittal sections showed much lower activities. The coronal section showed a high-intensity area in the superior lateral region of the lateral ventricle in the frontal lobe.

In contrast, the highlanders showed different brain activation patterns, as revealed in coronal and sagittal sections. Figure 3A is the horizontal section of highlanders showing similar high- (black arrowhead) and low-(white arrowhead) intensity areas as the lowlanders. The lateral sagittal section of highlanders did not show any significant intensified areas (Fig. 3B). The coronal section revealed a high-intensity area (Fig. 3C, black arrowhead) similar to that in the lowlanders in the frontal brain, but more medial and superior to the lateral ventricle. However, projection from this active area (Fig. 3C, black arrowhead) was not observed in the highlanders.

**Figure 4:**
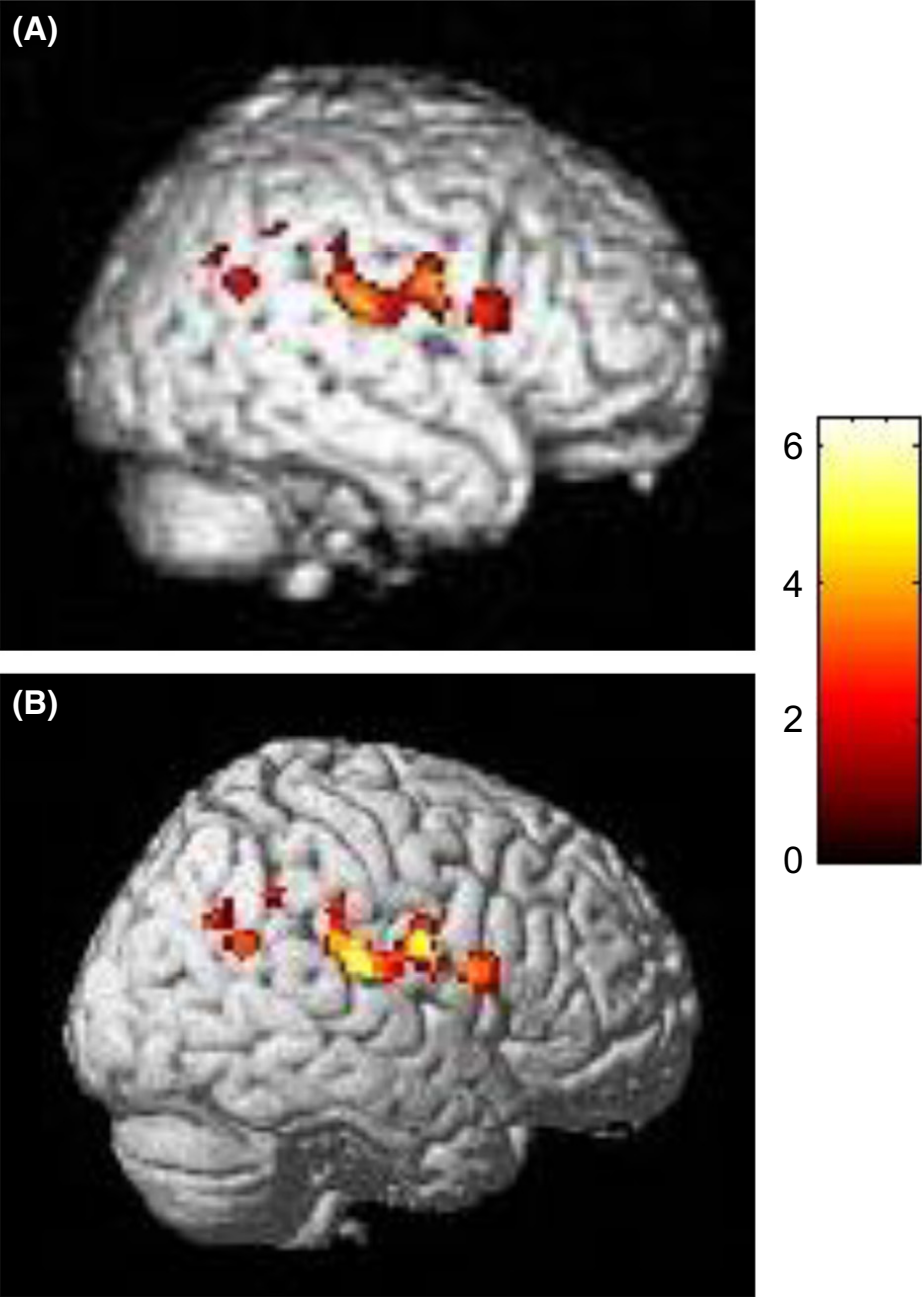
Lateral computer brain templates of overall active brain regions in (A) lowlanders and (B) highlanders. Larger and more intense areas were observed in the lowlanders, indicated by yellow over red colors (*P* < 0.001).

**Figure 5:**
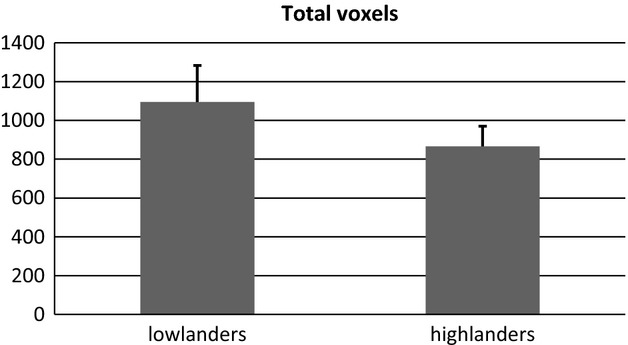
Comparison of total voxels in the brains of highlanders versus lowlanders upon mathematical calculation (*t*-test, *P* = 0.003). Bars shown are mean ± SD.

**Figure 6:**
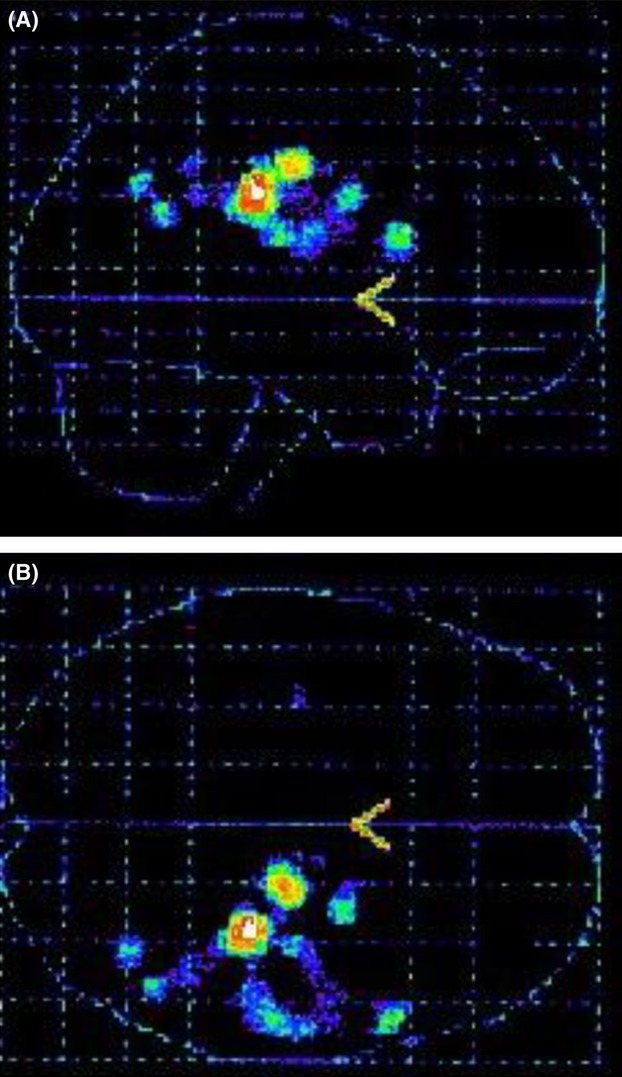
Computerized comparison of overall active brain regions between lowlanders and highlanders in (A) lateral and (B) horizontal views. Red and yellow areas present significant overlapping activated regions in both lowlanders and highlanders. Green and blue areas represent activated regions only present in lowlanders. Larger brain regions, and only in the right hemisphere, were involved in performing the mental calculation task in lowlanders than highlanders (*P* < 0.001).

The total activated areas in both lowlanders (Fig. 4A) and highlanders (Fig. 4B) were computed and expressed as voxels for comparison. The lowlanders showed an approximate 1.3× increase in voxels (Fig. 5) while working on this simple mental task when compared to the highlanders, and the lateral views on the brain templates of the two groups revealed larger activated areas in lowlanders than highlanders. A comparison of some of the active areas was shown in Figure 6A and B. The red and yellow areas indicated overlapping active areas shared by both lowlanders and highlanders. The green and blue areas were recorded in lowlanders only with *P* < 0.001. Greater areas in both deep frontal and parietal lobes were activated in lowlanders than highlanders (Fig. 6A). Figure 6B revealed that while the right hemisphere was primarily involved in performing the mental task. More active cortical regions were found in the lowlanders (blue and green areas) than the activated areas shared by both high and lowlanders (red and yellow areas).

## Discussion

Our results indicate that the parietal area is one of the major areas involved in mathematical computation as documented by others (Dehaene et al. 1999, 2003; Andres et al. 2012). In addition, the area in front of the executive motor strip, a part of the premotor area is also involved even in simple calculation in this study. It is likely that both the programing and association are necessary steps in performing the task. More importantly, the lowlanders and highlanders displayed subtle differences in the areas involved, indicating perhaps diversified brain functioning after adaptation of the highlanders upon centuries of evolution. Most interesting is perhaps that the highlanders could perform the same function of computing with fewer brain regions involved. This may be similar but not equal to athletes who were trained in high altitudes when returning to low levels exhibited better performance (Bailey and Davies 1997). Both lowlanders and highlanders in this study were submitted to full blood analysis and there was an increase in red blood cell count and a slight deviation in mature cell volume in the highlanders as predicted, while all other laboratory parameters being normal. The performance of the highlanders in mathematical calculation employing a smaller volume of the brain was not comparable to aging individuals who enlisted more volume of the brain to compensate for the same exercise when performed by young persons (Fang et al. 2005). The real mechanism of why highlanders could use smaller brain volume in a cognitive event has to be further explored.

## Conclusion

This study compared for the first time cognitive abilities and brain activation patterns of lowlanders (those native to an altitude of 1700 m above sea level) and highlanders (those native to an altitude of at least 3000 m above sea level) in performing a simple mental calculation task. Both lowlanders and highlanders successfully completed the task, but the latter group did so requiring the activation of significantly smaller brain regions. The findings added to the list of physiological changes demonstrated by individuals from high altitude, that is, exposed chronically to hypoxic environment. Future studies may be conducted in order to elucidate the underlying mechanisms of this characteristic of the highlanders.
